# Optimization of Scat Detection Methods for a Social Ungulate, the Wild Pig, and Experimental Evaluation of Factors Affecting Detection of Scat

**DOI:** 10.1371/journal.pone.0155615

**Published:** 2016-05-25

**Authors:** David A. Keiter, Fred L. Cunningham, Olin E. Rhodes, Brian J. Irwin, James C. Beasley

**Affiliations:** 1 Savannah River Ecology Laboratory, University of Georgia, Aiken, South Carolina, United States of America; 2 D. B. Warnell School of Forestry and Natural Resources, University of Georgia, Athens, Georgia, United States of America; 3 National Wildlife Research Center, United States Department of Agriculture Animal and Plant Health Inspection Service, Starkville, Mississippi, United States of America; 4 Odum School of Ecology, University of Georgia, Athens, Georgia, United States of America; 5 U. S. Geological Survey, Georgia Cooperative Fish and Wildlife Research Unit, D. B. Warnell School of Forestry and Natural Resources, University of Georgia, Athens, Georgia, United States of America; University of Sydney, AUSTRALIA

## Abstract

Collection of scat samples is common in wildlife research, particularly for genetic capture-mark-recapture applications. Due to high degradation rates of genetic material in scat, large numbers of samples must be collected to generate robust estimates. Optimization of sampling approaches to account for taxa-specific patterns of scat deposition is, therefore, necessary to ensure sufficient sample collection. While scat collection methods have been widely studied in carnivores, research to maximize scat collection and noninvasive sampling efficiency for social ungulates is lacking. Further, environmental factors or scat morphology may influence detection of scat by observers. We contrasted performance of novel radial search protocols with existing adaptive cluster sampling protocols to quantify differences in observed amounts of wild pig (*Sus scrofa*) scat. We also evaluated the effects of environmental (percentage of vegetative ground cover and occurrence of rain immediately prior to sampling) and scat characteristics (fecal pellet size and number) on the detectability of scat by observers. We found that 15- and 20-m radial search protocols resulted in greater numbers of scats encountered than the previously used adaptive cluster sampling approach across habitat types, and that fecal pellet size, number of fecal pellets, percent vegetative ground cover, and recent rain events were significant predictors of scat detection. Our results suggest that use of a fixed-width radial search protocol may increase the number of scats detected for wild pigs, or other social ungulates, allowing more robust estimation of population metrics using noninvasive genetic sampling methods. Further, as fecal pellet size affected scat detection, juvenile or smaller-sized animals may be less detectable than adult or large animals, which could introduce bias into abundance estimates. Knowledge of relationships between environmental variables and scat detection may allow researchers to optimize sampling protocols to maximize utility of noninvasive sampling for wild pigs and other social ungulates.

## Introduction

Noninvasive sampling, such as the collection of fecal material, by definition, allows wildlife researchers to obtain information about a population of interest while minimizing potential disturbances [1]. Collection of fecal samples is one of the most frequently used noninvasive sampling techniques, and has been applied extensively in studies of animal diet [2], disease prevalence [3], endocrinology [4], phylogeography, genetics, and population ecology [5]. In particular, noninvasive genetic sampling is increasingly being used in a capture-mark-recapture framework to estimate population size or density, due to reduced impacts to target species and logistical or financial benefits over traditional capture-recapture approaches [6,7]. However, genetic capture-mark-recapture studies typically require large sample sizes because samples can experience high levels of DNA degradation [8,9]. Therefore, it is generally recommended that researchers collect 2.5–3 times as many samples as the suspected number of animals present in the sampling area to yield robust estimates of population size [10]. The difficulty in obtaining sufficient sample sizes of feces to estimate abundance may be further exacerbated in species with relatively low defecation rates (e.g. wild boar *Sus scrofa*; [11]). For these reasons, optimization of sampling protocols to maximize the number of sampled scats and efficiency of collection methods is needed to allow sampling of large spatial areas.

In many types of research utilizing feces, collected scat samples should be representative of the population of scats as a whole, which supports the use of random sampling techniques, such as transects. However, these techniques can be inefficient at encountering sufficiently large numbers of samples due to the non-random distribution of scats on the landscape [2]. For this reason, researchers frequently collect scat in locations where density of scat is likely to be high based upon the behavior of the target taxa. For carnivores, this is often along roads and trails [12,13] or at communal latrines sites [14,15]. While much research has been performed to optimize fecal sampling methods for carnivores [16,17], further optimization is necessary for social ungulates, where transect sampling methods may be inadequate to obtain sufficient sample sizes of scat [9]. Unlike carnivores, many ungulate species avoid roads [18] and do not form latrine sites (but see [19,20]), requiring additional effort to acquire sufficient sample sizes of scats. The deposition of scat by many herbivores may, however, be greatly influenced by the presence of social structures, such as aggregative behaviors.

In an effort to account for the social behavior of some ungulate species, Ebert *et al*. [21] modified transect sampling to include elements of adaptive cluster sampling [22] to increase the number of scats encountered in a genetic capture-mark-recapture study of wild boar. In their methodology, a 5-m radius was searched around each scat encountered along a linear transect and a new 5-m radius was searched for each subsequent scat encountered, until no further scats were detected [21]. This protocol, hereafter referred to as ACS, is intended to take advantage of the deposition of scat by groups of animals; however, the effectiveness and efficiency of ACS depends on the distribution of scat on the landscape. For instance, ACS will not detect scat falling beyond the defined search radius, and its sequential search structure could lead researchers along highly directional search paths, suggesting that an adaptive cluster sampling approach incorporating a single larger search radius could be more effective.

Beyond accounting for the distribution of scat on the landscape through sampling techniques, knowledge of factors that may affect detection of scat can aid researchers in the design of sampling protocols to maximize sample collection. Because humans primarily detect scat visually, as opposed to scat detection dogs, which primarily use olfaction, it is likely that some environmental characteristics, such as percentage of vegetative ground cover, affect the detectability of scat by increasing visual obstruction. Further, characteristics of the scat itself, such as size and number of fecal pellets, could affect detection of scat samples. Knowledge of these relationships is necessary for the development of an appropriate sampling design and may aid researchers in assessing whether sufficient samples can be collected to meet research objectives.

We selected wild pigs (*Sus scrofa*) as a model organism to evaluate the effects of sampling protocol, scat characteristics, and environmental attributes on detection rates of scats because pigs exhibit numerous characteristics representative of a number of non-carnivorous species to which these methods could potentially be applied. For example, this species exhibits relatively low defecation rates [9,11], and, like many non-carnivorous mammals, infrequently deposits scats on roads. Thus, fecal sampling for wild pigs can be challenging because of the low encounter and detection probabilities associated with their scat. Furthermore, similar to many other ungulates, wild pigs often travel in small groups [23], and frequently deposit scat in clusters, though they do not create latrine sites. As a practical matter, wild pigs inflict tremendous damage on native ecosystems and pose a risk to humans, livestock, and wildlife populations as a reservoir for transmissible diseases (see [24–26] for review), necessitating the optimization of fecal sampling techniques for use in estimating abundance of this invasive species.

Our objectives in this study were 1) to compare scat detection rates using the 5-m radius ACS search protocol to a novel series of fixed-area radial search protocols (5, 10, 15, and 20 m) in two habitat types suspected to contain different densities of wild pigs to elucidate differences in the number of fecal samples encountered by each method, and 2) to evaluate the effects of habitat, weather, and scat characteristics on the detectability of scat by human observers. We hypothesized that larger-sized radial searches (15 or 20 m) would result in higher rates of scat encounters than the ACS sampling technique in both the high and low wild pig density study sites and that scat detectability would decrease as the percentage of vegetative ground cover increased and the number of fecal pellets present (single vs. group of three) and size of fecal pellets decreased. The characteristics affecting scat detection measured in this study are widely applicable across ecoregions and species, allowing broad application of these relationships. Thus, this information will provide researchers with a foundation to optimize sampling protocols for future studies requiring the collection of scats in social ungulates.

## Methods

### Study Area

**Fig 1:**
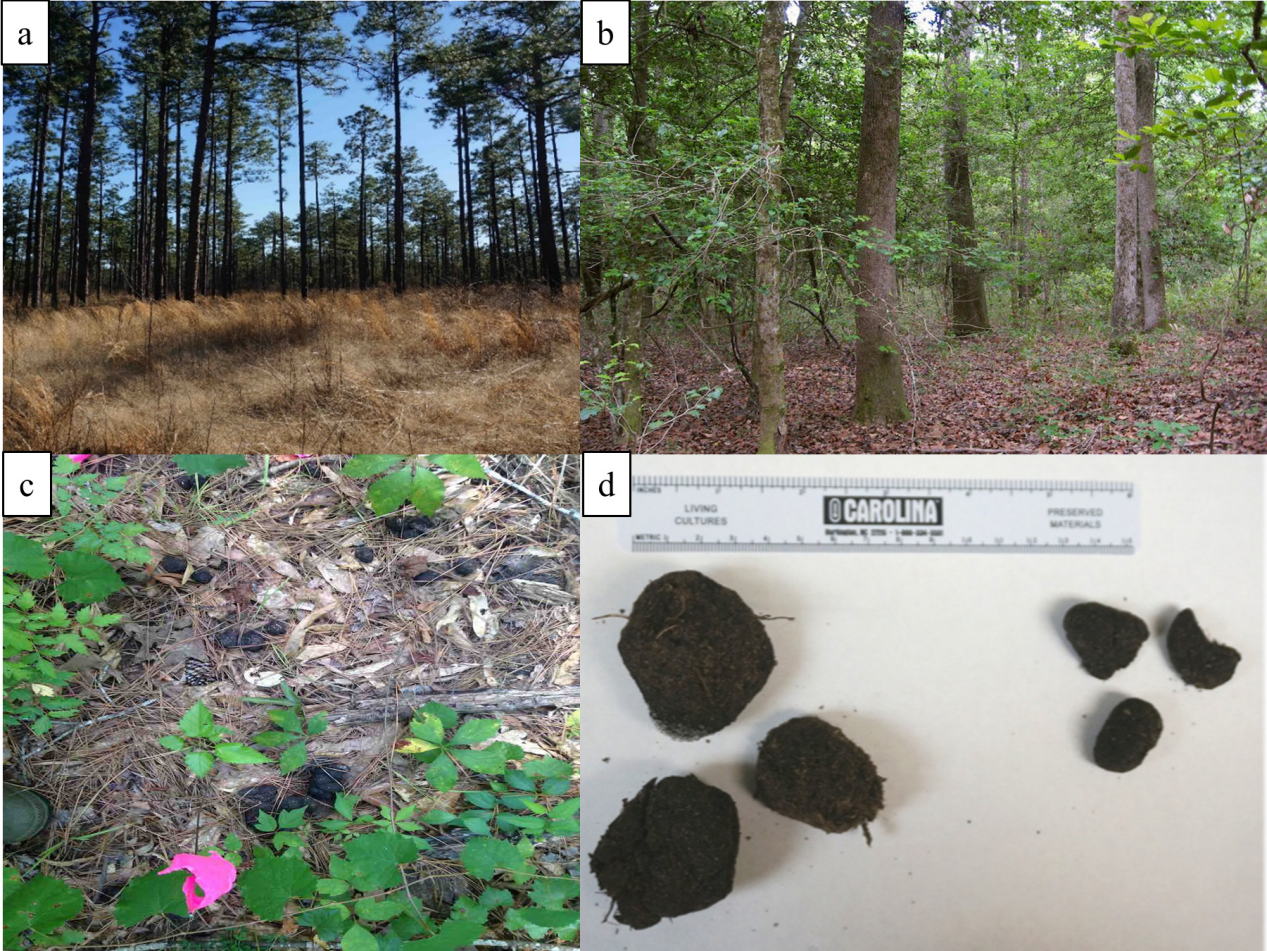
Examples of common habitat types of the Savannah River Site, South Carolina, USA, and wild pig feces. Examples show (**a.**) upland pine habitat, (**b.**) bottomland hardwood habitat, (**c.**) *in situ* wild pig (*Sus scrofa*) scat and vegetative ground cover, and (**d.**) wild pig scat distinguished into large (left) and small (right) size categories for experimental evaluation.

We compared search protocols to detect scat at the Savannah River Site (SRS), a 78,000 ha United States Department of Energy facility on the Georgia-South Carolina border (33°20’N, 81°44W) in the Upper Coastal Plain physiographic region of the United States. Approximately 68% of habitat on the SRS consists of upland pine (Fig 1A), mainly loblolly pine (*Pinus taeda*), long-leaf pine (*P*. *palustris*), and slash pine (*P*. *elliotii*) managed by the United States Forest Service [27,28]. Common understory plants in upland pine habitat at the SRS include broom sedge (*Andropogon virginicus*), bracken fern (*Pteridium aquilinum*), poison oak (*Toxicodendron pubescens*), deerberry (*Vaccinium stamineum*), sparkleberry (*Vaccinium arboreum*), wax myrtle (*Morella cerifera*), sweetgum (*Liquidambar styraciflua*), and scrub oaks (*Quercus* spp.) [28]. An additional 22% of the SRS consists of swamp and riparian bottomland habitat (Fig 1B) dominated by water-oak (*Quercus nigra*), tulip-poplar (*Liriodendron tulipifera*), sweetgum, and maple (*Acer* spp.; [28,29]). Understory plants in the bottomland hardwood area include switchcane (*Arundinaria tecta*), redbay (*Persea palustris*), shining fetterbush (*Lyonia lucida*), American holly (*Ilex opaca*), and dwarf palmetto (*Sabal minor*) [28]. Greater detail on the vegetative communities associated with these habitat types can be found in [28]. Elevation of the SRS ranges from 30–115 m above sea level. When the SRS was closed to the public in 1952 and resident farmers moved offsite, large numbers of domestic pigs remained. These animals since reverted to a feral state and expanded in abundance and distribution throughout the area [30], and today this population shows signs of introgression by wild boar genetics; for this reason we refer to them as wild pigs [31]. Previous research has suggested that wild pigs prefer bottomland hardwood habitat to upland pine habitat at the SRS [29,30], leading us to suspect that wild pig densities are different between these habitat types.

Additional experimental trials to evaluate detection rates of scat were conducted at Whitehall Forest, a 304 ha property owned by the University of Georgia, D.B. Warnell School of Forestry and Natural Resources. This property is near the city of Athens in the Piedmont region of Georgia (33°56’N, 83°24’W), USA. The upland portion of Whitehall forest where this research took place is characterized by loblolly pine, shortleaf pine (*P*. *echinata*), oak, sweetgum, and hickory (*Carya* spp., [32]). Understory vegetation in Whitehall Forest is similar to that described in [33] and includes sweetgum, oak, muscadine (*Vitis rotundifolia*), and wingstem (*Verbesina alternifolia*).

### Data Collection

#### Search Protocol Comparisons

We conducted scat sampling along 22 transects at the SRS from 14 July– 7 August, 2014. Transects were spaced approximately 0.5 km apart and oriented roughly north to south. We used a handheld GPS unit to mark a beginning and ending point for each transect on a pair of parallel roads in the habitat type of interest, and searched the area between the two points for wild pig scat. Researchers were able to search a width of approximately 3 m: 1.5 m on either side of each transect. In bottomland hardwood habitat, we sampled 20.8 km of transects with an average length of 2.97 km per transect (*SE* = 0.60). In upland pine habitat, we sampled 36.9 km of transects with an average length of 2.46 km per transect (*SE* = 0.15).

Each time we encountered scat on a transect, we sequentially applied each of the five search protocols being compared. The first protocol used was the ACS method employed by Ebert *et al*. [21] in which we searched a 5-m radius around the initial scat encountered on the transect and then searched a 5-m radius around each additional scat encountered within the 5-m radius, and each subsequently encountered scat, until no further scat were found. The remaining protocols each consisted of a single search radius (either 5, 10, 15, or 20 m), centered around the scat detected within the original search window (approximately 3 m) along the main transect. These single search protocols did not include additional radii around scat subsequently found off-transect within the search radius. We marked each scat encountered with a survey flag to prevent double counting within each protocol and recorded the distance and bearing of each additional scat found to the initial scat encountered. As all five sampling protocols were applied each time a scat was found, we assumed that the vegetative cover would remain relatively constant across the protocols at each scat cluster (i.e. would not vary consistently between a 15 m and a 20 m radius centered on the same point), and therefore would not impact comparison of protocol effectiveness. For this reason we did not measure the percentage of vegetative ground cover at each encountered scat in this portion of the study.

#### Detection Probabilities of Scat

To estimate detection rates of scat, we created four 100 m transects in mixed pine-hardwood habitat at Whitehall Forest. Transects were spaced approximately 25 m apart, delineated by marked survey flags placed at 5 m intervals, and oriented roughly southeast to northwest. Wild pigs are not established at Whitehall Forest, so we added previously collected wild pig scat from the SRS to form experimental transects with known scat locations. The overall location for these transects was selected for its variable amounts of vegetative cover in order to test for an effect of percent vegetative ground cover on the probability of scat detection (Fig 1C).

We visually separated previously collected wild pig scats into 2 size classes (SIZE): small and large (Fig 1D), because previous research has demonstrated that scat from smaller species may be less detectable than that of larger species [34]. To ensure that our visual classification represented distinct size classes, we measured a sub-sample of 10 randomly selected fecal pellets from each to determine an average volume for fecal pellets of the small ($\bar{x}$ *=* 5.98 cm^3^, *SE* = 1.17) and large size class ($\bar{x}$ = 27.79 cm^3^, *SE* = 2.06). Experimental scat treatments were created by dividing fecal pellets of the 2 size classes into either single (consisting of one fecal pellet) or group categories (consisting of 3 fecal pellets; NUMB). We chose to use 3 fecal pellets for the group category, as this number was determined to reasonably represent conditions we observed in the field based on pilot studies conducted on the SRS. We placed 4–5 scats of each of the 4 treatments (small-single, small-group, large-single, large-group) at randomly generated distances along each transect for a total of 18–20 scat locations per transect. We randomly assigned each scat to be placed within 1 m to the left or right of each transect to ensure that all placements were within the estimated effective search distance for pig scat along the transect. We used a 1 m^2^ area framed by PVC pipe and gridded into 100 equal-sized cells to estimate the percentage of vegetative ground cover (COVER) present at each location that a scat was placed.

From 17–20 November, 2014, 56 student volunteers, from a University of Georgia wildlife techniques course, sampled the four constructed transects. Each volunteer only walked an individual transect once and recorded each distance at which they detected a scat. This resulted in a binomial capture history for every known scat location consisting of its detection or non-detection by each observer that sampled each transect. Before sampling began each day, we walked transects and replaced any missing scats with scats of the same treatment type. Prior to sampling, volunteers were instructed that number and size of fecal pellets might vary and were shown examples of wild pig scat. None of the volunteers had prior experience searching transects for scat. One of the sampling periods occurred immediately following a rainstorm, so we incorporated rain prior to sampling (RAIN) as an additional predictor variable (event categorized as a 1 for rain and a 0 for non-rain). No permits were required for this work as researchers and volunteers did not come into contact with live or dead animals.

### Data Analysis

#### Search Protocol Comparison

The negative binomial distribution is often appropriate for modeling non-negative, discrete count data [35], and thus, we developed a linear mixed model, in which the observed scat counts followed a negative binomial distribution. Sampling protocol (i.e., ACS and 5-, 10-, 15-, and 20-m radial searches) was included in the model as a categorical fixed-effect predictor variable to determine whether significant differences existed in the number of scat found among the different search protocols. We also evaluated a model incorporating the broad category of habitat type as an additional fixed effect to determine whether habitat type affected the number of scats found. Individual transects were treated as normally-distributed random effects in each of these models. Analyses were conducted using packages *lme4* and *glmmADMB* in R [36]. We judged the relative support of models using the second order Akaike’s Information Criterion (AICc) and AIC weight (AICw_i_), a measure of model likelihood [37]. If models were ≤ 2.0 AICc units from the best model, we considered them to be supported [37] unless they were judged to contain an uninformative parameter [38]. A model with an uninformative parameter is defined as being within 2.0 AICc units of the best model, with only one additional parameter and a similar model deviance [38].

#### Detection Probabilities of Scat

We created 5 *a priori* models (Table 1) to predict the probability of detecting or failing to detect scat, based on expected relationships between our predictor variables of interest (percent vegetative ground cover, scat pellet size, number of fecal pellets, and whether rain occurred immediately prior to sampling) and the observed response variable (detection or non-detection of wild pig scat). In these models, the predictor variables listed above were included as fixed effects, while observer and transect were held as random effects. The collected observations of “failure” and “success” of detecting scat were assumed to be binomially distributed. We used the same information-theoretic metrics as above to compare these 5 candidate models. All data and code are available online (S1 and S2 Tables, S1 and S2 Files).

**Table 1:** Model selection results for *a priori* models relating probability of detecting wild pig scat to predictor variables, Whitehall Forest, Georgia, USA, 2014.

Model	K^a^	AICc	ΔAICc	*w*_*i*_	-LL^b^
**NUMB**^**c**^**+SIZE**^**d**^**+COVER**^**e**^**+RAIN**^**f**^	7	4852.42	0.00	0.95	-2419.19
**SIZE+COVER+RAIN**	6	4858.57	6.15	0.04	-2423.27
**NUMB+SIZE+COVER**	6	4865.86	13.44	0.00	-2426.92
**NUMB+SIZE**	5	4875.76	23.34	0.00	-2432.87
**NUMB**	4	4982.13	129.71	0.00	-2487.06

Models are ranked by change in second order Akaike’s Information Criterion (ΔAICc) and AIC weight (*w*_*i*_*)*

^a^ Number of parameters including two random-effect variances (Observer and Transect) and a global intercept term

^b^ Negative log-likelihood

^c^ Number of fecal pellets (single or group)

^d^ Size of fecal pellets (small or large)

^e^ Percentage of vegetative ground cover

^f^ Rain event immediately prior to sampling (event or non-event)

## Results

### Search Protocol Comparison

**Fig 2:**
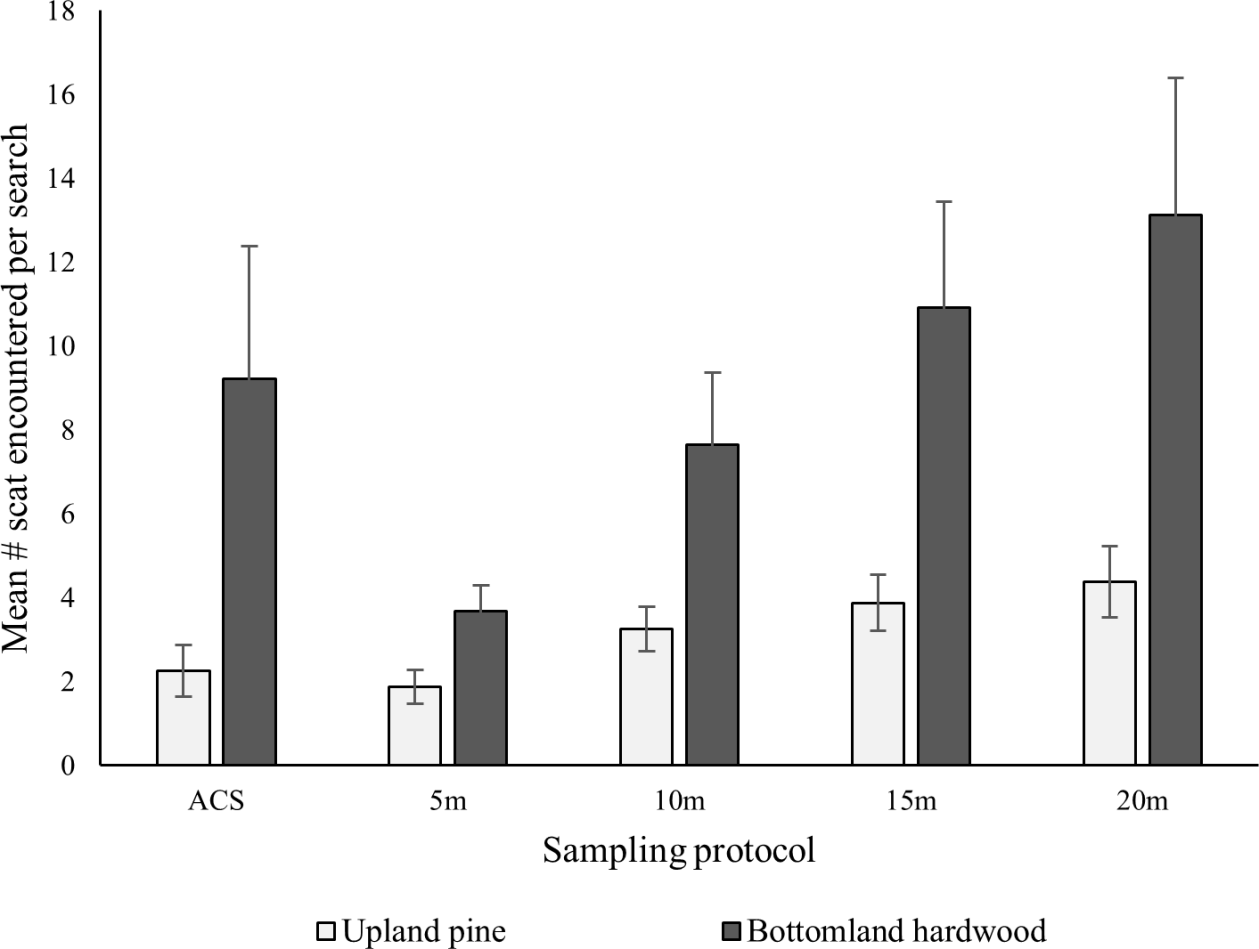
Amount of wild pig scat encountered by observers along transects at the Savannah River Site, South Carolina, USA, 2014. Depicts mean number of scats detected per cluster by the adaptive cluster sampling (ACS) scat sampling protocol and 5-, 10-, 15-, and 20-m radial search protocols in bottomland hardwood (BH) and upland pine (UP) habitat. Error bars represent one standard error.

In total, we encountered 35 scats in 8 clusters in the upland pine habitat and 467 scats in 33 clusters in the bottomland hardwood habitat of the SRS. Thus, we found a higher average scat density in the bottomland hardwood habitat ($\bar{x}$ = 73.71 scats/km, *SE* = 48.54) than in upland pine habitat ($\bar{x}$ = 0.94 scats/km, *SE* = 0.41). The negative binomial mixed model of the number of scats encountered that incorporated habitat type and sampling protocol as fixed effects was more supported (**Δ**AICc = 0.00, AIC*w*_*i*_. = 0.80) than the model that did not incorporate habitat type (**Δ**AICc = 2.58, AIC*w*_*i*_ = 0.20), suggesting that the efficacy of sampling differs between habitat types. Our most supported model indicated that fewer scats were found in upland pine than bottomland hardwood habitat, as we hypothesized (Fig 2). We believe that this result was likely due to differing use of habitats by wild pigs (Kurz and Marchinton 1972), but it is possible that detectability of scat in the two habitat types differed as well.

**Fig 3:**
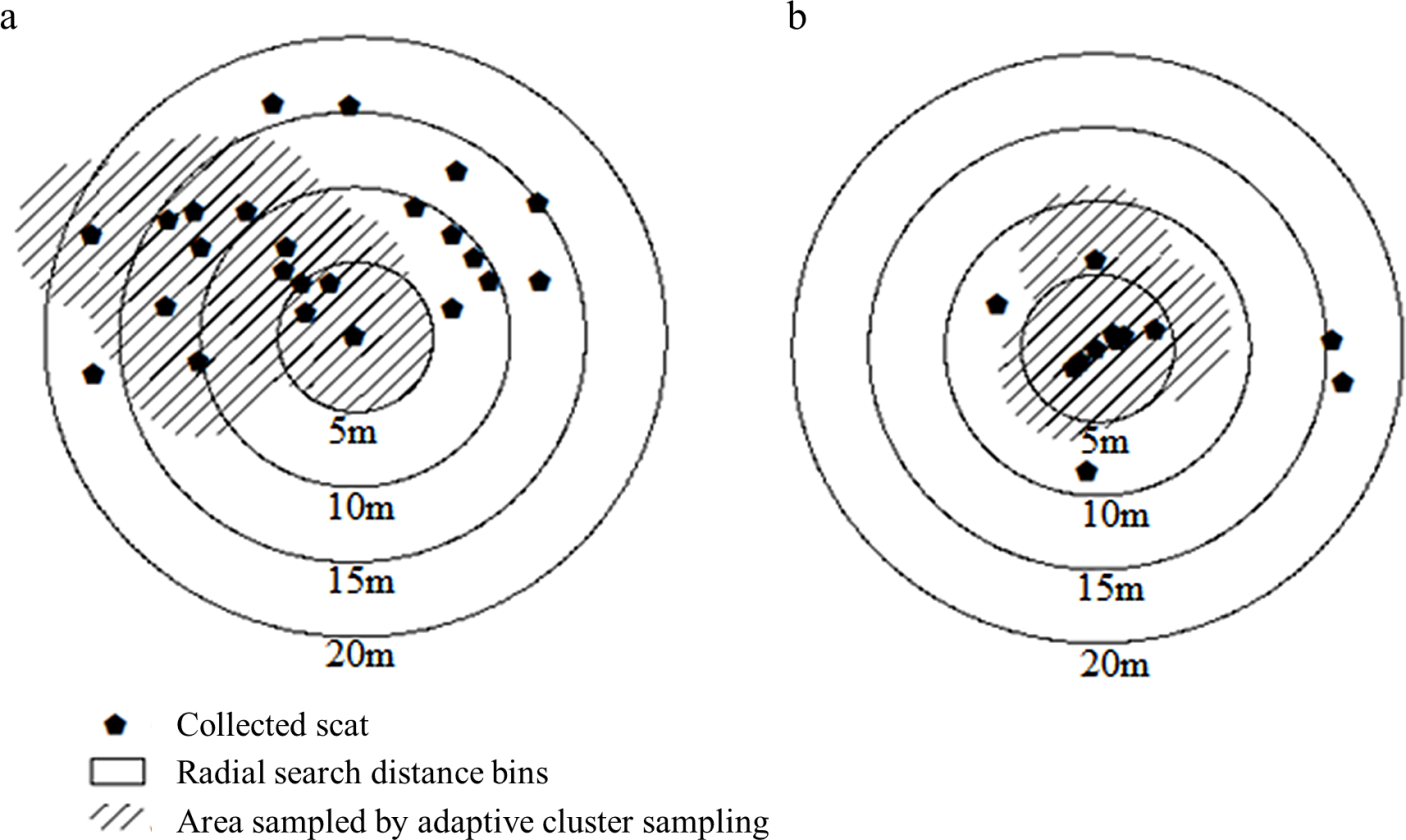
Illustration of tested scat sampling protocols. Diagrams illustrate differences in the number of wild pig scats encountered by the adaptive cluster sampling (ACS, shown as shaded area) protocol and 5-m, 10-m, 15-m, and 20-m radial search protocols in areas of high (a.) and low density (b.) of scat. Diagrams represent actual spatial distributions of scats observed in bottomland hardwood habitat at the Savannah River Site, South Carolina, USA, 2014.

The ACS method ($\bar{x}$ = 7.85, *z* = 6.79, *SE* = 2.58) resulted in a larger number of detected scats than a 5-m radial search ($\bar{x}$ = 3.32, *SE* = 0.52, *z* = -2.83, *P <* 0.010), but no difference in mean count was detected between ACS and the 10-m radius search ($\bar{x}$ = 6.78, *SE* = 1.41, *z* = 0.46, *P* = 0.648). Significantly more scats were encountered using the 15- and 20-m radius searches ($\bar{x}$ = 9.54, *SE* = 2.08, *z* = 2.33, *P* = 0.019 and $\bar{x}$ = 11.41, *SE* = 2.69, *z* = 3.20, *P* < 0.010 respectively, Fig 3, Table 2) than for ACS. As might be expected, the amount of time required to implement a single search increased with the amount of area searched (e.g. more time was needed to search a 10 m radius than a 5 m radius). When the amount of area searched is not fixed, as is the case with the ACS protocol we tested, it is not possible to compare the amount of time a search will take to fixed area protocols, as the time required varies depending on the distribution of scats and the amount of spatial overlap between searches.

**Table 2:** Parameter estimates for the best model comparing scat sampling protocols at the Savannah River Site, South Carolina, USA, 2014.

Parameter	β	SE	95% CI
**Reference (adaptive cluster sampling, bottomland hardwood habitat)**	1.845	0.272	1.32 to 2.38
**5-m radius**	-0.533	0.188	-0.90 to -0.16
**10-m radius**	0.080	0.176	-0.26 to 2.18
**15-m radius**	0.401	0.172	0.06 to 0.74
**20-m radius**	0.545	0.17	0.21 to 0.88
**Upland pine habitat**	-0.918	0.389	-1.68 to -0.16

Table reports parameter estimates (β), standard errors, and 95% confidence intervals for the parameters of a negative binomial mixed model of the effects of habitat type and sampling protocol on the number of wild pig scats detected.

### Detection Probabilities of Scat

**Fig 4:**
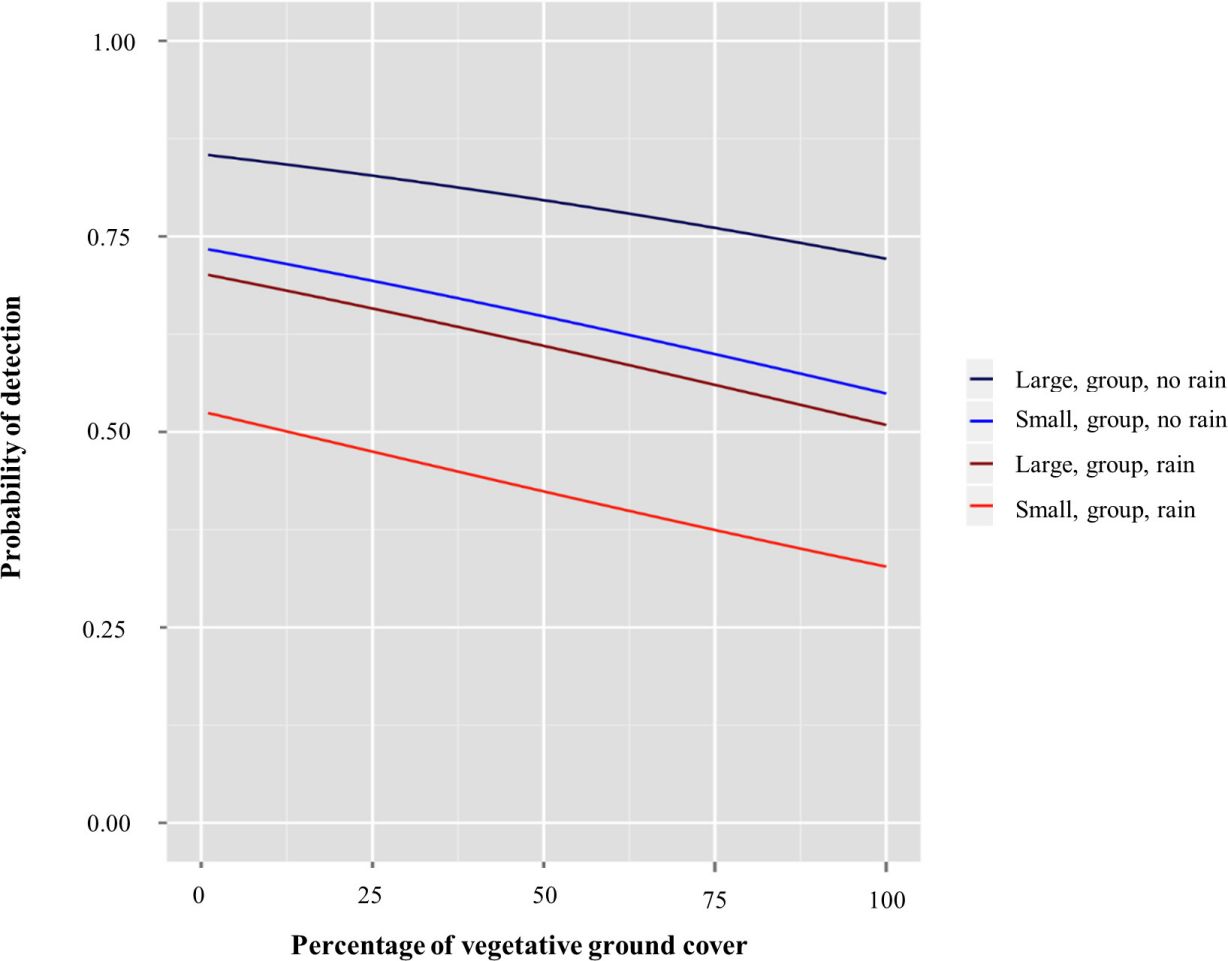
Relationships between environmental and scat characteristics and detectability of wild pig scat, Whitehall Forest, Georgia, USA, 2014. Graph depicts predicted probability of detection for groups of wild pig scat as a function of percent vegetative ground cover, fecal pellet size, and whether rain occurred immediately prior to sampling.

Each of the 4 experimental transects was sampled on average 31.3 times (SE = 0.18) by volunteers. Among the 56 volunteers, the percentage of scats detected on a single transect was highly variable, ranging from 5.2%– 85.0%. The most supported model of scat detectability revealed that the percentage of vegetative ground cover, scat pellet size (small or large), number of fecal pellets (single or group of three), and whether rain occurred prior to sampling (event or non-event) were important predictors of scat detection (ΔAICc = 0.00, AIC*w*_*i*_ = 0.95, Table 1, Table 3). As percent ground cover increased, the probability of detecting scat decreased (Fig 4, Table 3). Smaller-sized scats had a lower detectability than larger-sized scats, and scat groups had higher detectability than single pellets (Table 3). Rain prior to sampling also noticeably reduced the probability of scat detection by observers (Fig 4, Table 3). As might be expected, the estimated remaining variability among the four replicate transects (0.034) was small relative to the estimated variability among volunteer observers (0.526).

**Table 3:** Parameter estimates for the best model of factors affecting detectability of wild pig scat at Whitehall Forest, Georgia, USA, 2014.

Parameter	β	SE	95% CI
**Reference (large, group, ground cover = 0, no rain)**	1.776	0.172	1.439 to 2.113
**Single Pellet**	-0.204	0.071	-0.343 to -0.065
**Small Size**	-0.755	0.072	-0.896 to -0.614
**Ground Cover**	-0.008	0.002	-0.012 to -0.004
**Rain**	-0.916	0.216	-1.339 to -0.493

Table reports parameter estimates (β), standard errors, and 95% confidence intervals for the best binomial mixed model of probability of scat detection as a function of fecal pellet number, pellet -size, percent vegetative ground cover, and whether rain occurred prior to sampling.

## Discussion

In previous noninvasive genetic studies of *Sus scrofa* and other mammals, obtaining a sufficient sample size of feces has been a primary limiting factor to developing robust mark-recapture estimates [9, 39]. Sample size also can limit possible inferences of research on diet, disease prevalence, endocrinology, and other investigations utilizing scat. We demonstrated that use of a fixed radius (15 or 20 m) sampling area around the initial scat encountered along a transect was more effective in increasing sample size than the ACS protocol previously used for the collection of samples to estimate the population size of *Sus scrofa* [21]. In theory, ACS could encounter more scat than a fixed radial search in areas of uniformly high scat density, although such situations likely occur infrequently in nature. Moreover, the area searched by ACS is entirely dependent upon the spatial arrangement of scat present because every encountered scat prompts a new area to be searched, which could lead to an exceptionally large area sampled by this method, and, therefore, a large time required for a single search. Despite this, our fixed radial search method was more effective in encountering scat samples than ACS, even in areas of high scat density (i.e., 73.71 scats/km in bottomland hardwood habitat). Therefore, a radial search method as applied in this study appears to result in an increased sample size of collected scats for wild pigs, increasing the probability of successful estimation of abundance or density. Further exploration into these methods may be warranted for other social species.

Our research also revealed that both scat size and number of fecal pellets present affected detection of scat by observers. Juvenile wild pigs generally produce smaller scat than adults, and if smaller scats are less detectable, as demonstrated in this study, and the difference is not accounted for, biased detection rates could result in inaccurate estimates of population size. Though we tested only two specific size categories of scat, we expect that the relationship between probability of detection and scat size might vary more generally, similar to that of carnivore species [34]. Likewise, individual animals may generally be less detectable by scat surveys than those traveling in groups, as an individual animal will produce fewer fecal pellets than a group of animals. These effects of scat size and social structure on detectability could reduce accuracy in estimates of population size when using genetic capture-mark-recapture methods, but could be accounted for in the development of mark-recapture models.

As we hypothesized, increased vegetative ground cover resulted in decreased detection of scats by observers, most likely as a result of visual obstruction. This suggests that when possible, researchers may want to design surveys to take advantage of habitat types and seasons in which ground cover will be reduced to maximize the collection of fecal samples. Rain immediately prior to sampling also reduced the probability of an observer detecting scat. Research has demonstrated that the type of substrate present can affect detectability of animal sign by observers [34,40]. It seems likely that, in this study, rain reduced visual contrast between the scat and local substrate, diminishing the detectability of scats by observers. Exposure to rainfall and wet conditions also decreases the chances of successful genetic analysis of fecal samples [41,42]. Therefore, researchers might avoid sampling immediately following rain to maximize the number and quality of scats collected.

Overall, our fixed radius method was able to detect more fecal samples than ACS, when both methods were applied to the same locations. Increased sample collection may aid in improving abundance estimates of wild pigs and other social mammals through increased accuracy and precision and reduced bias [6,43]. Higher capture probabilities, which often result from increased sample sizes, also allow better detection of individual heterogeneity in abundance estimation [44]. Many species exhibit defecation patterns similar to wild pigs in that their defecation on roads is infrequent and they do not habitually create and use latrine sites, thereby eliminating two common sources of scat samples [2]. Therefore, search protocols such as those outlined in this paper should be useful for improving the performance of scat surveys for many social ungulates, in which sampling of roads or latrine sites is generally insufficient or infeasible. Knowledge of the relationships between scat detectability by observers and environmental and scat characteristics should be used in conjunction with information about the behavioral ecology of the taxa of interest to develop taxa-specific sampling protocols to meet targeted sample sizes.

## Supporting Information

S1 File R code for comparison of scat sampling protocols. (R)

S2 File R code for evaluation of factors affecting detectability of wild pig scat. (R)

S1 Table Data for comparison of scat sampling protocols at the Savannah River Site, Aiken, South Carolina, USA, 2014. (CSV)

S2 Table Data for evaluation of factors affecting detectability of wild pig scat, Whitehall Forest, Georgia, USA, 2014. (CSV)
